# The thiol-disulfide exchange activity of *At*PDI1 is involved in the response to abiotic stresses

**DOI:** 10.1186/s12870-021-03325-7

**Published:** 2021-11-23

**Authors:** Ying Lu, Li Yuan, Zhou Zhou, Mengyu Wang, Xiaoyun Wang, Shizhong Zhang, Qinghua Sun

**Affiliations:** 1 College of Life Science, State Key Laboratory of Crop Biology, Shandong Agricultural University, Taian, Shandong 271018 People’s Republic of China; 2 Institute of Shandong River Wetlands, Jinan, Shandong 271100 People’s Republic of China

**Keywords:** *At*PDI1, *Arabidopsis*, Abiotic stress, ABA signaling, ROS elimination

## Abstract

**Background:**

*Arabidopsis* protein disulfide isomerase 1 (*At*PDI1) has been demonstrated to have disulfide isomerase activity and to be involved in the stress response. However, whether the anti-stress function is directly related to the activities of thiol-disulfide exchange remains to be elucidated.

**Results:**

In the present study, encoding sequences of *At*PDI1 of wild-type (WT) and double-cysteine-mutants were transformed into an *At*PDI1 knockdown *Arabidopsis* line (*pdi*), and homozygous transgenic plants named *pdi*-*At*PDI1, *pdi*-*At*PDI1_m1_ and *pdi*-*At*PDI1_m2_ were obtained. Compared with the WT and *pdi*-*At*PDI1, the respective germination ratios of *pdi*-*At*PDI1_m1_ and *pdi*-*At*PDI1_m2_ were significantly lower under abiotic stresses and exogenous ABA treatment, whereas the highest germination rate was obtained with *At*PDI1 overexpression in the WT (WT- *At*PDI1). The root length among different lines was consistent with the germination rate; a higher germination rate was observed with a longer root length. When seedlings were treated with salt, drought, cold and high temperature stresses, *pdi*-*At*PDI1_m1_, *pdi*-*At*PDI1_m2_ and *pdi* displayed lower survival rates than WT and *At*PDI1 overexpression plants. The transcriptional levels of ABA-responsive genes and genes encoding *ROS-quenching* enzymes were lower in *pdi*-*At*PDI1_m1_ and *pdi*-*At*PDI1_m2_ than in *pdi*-*At*PDI1.

**Conclusion:**

Taken together, these results clearly suggest that the anti-stress function of *At*PDI1 is directly related to the activity of disulfide isomerase.

**Supplementary Information:**

The online version contains supplementary material available at 10.1186/s12870-021-03325-7.

## Background

As a thiol-disulfide oxidoreductase, protein disulfide isomerase (PDI) catalyzes the oxidative folding and isomerization of nascent polypeptide chains [1–3]. Some PDIs also display chaperone or anti-chaperone activity [4, 5]. Typical PDI, for example, human PDI (*h*PDI), contains two thioredoxin-like domains, *a* and *a′*, linked by domains *b* and *b′* [6]. The enzymatic activities of PDI are directly related to the CXXC motif in the *a* and *a′* domains, respectively, and mutations of the conserved cysteines in the motifs lead to loss of enzymatic activities [7–9]. Extensive investigations have demonstrated that animal PDIs, such as *h*PDI, are located in the endoplasmic reticulum (ER) with a carboxyl-terminal KDEL sequence as the retention signal [6]. Under adverse conditions, the process of correct protein folding is disturbed, which leads to ER stress and the unfolded protein response (UPR), finally resulting in cell death [10]. When the human body is threatened by pathogens, the expression of PDIs is upregulated, keeping unfolded or misfolded proteins correctly folded with functional conformation [11–14]. Furthermore, investigations demonstrated that inhibition of *h*PDI caused the accumulation of unfolded or misfolded proteins [15, 16]. Generally, the formation of a native conformation of the peptide chain occurs with the help of molecular chaperones and is then oxidized to form disulfide bonds. When the activity of oxidoreductase was blocked, *h*PDI could also act as a chaperone, binding to misfolded or unfolded proteins and facilitating proper folding to mitigate the damage under stress conditions, which means that the chaperone activity is independent of its redox active site [17].

Homologous proteins of *h*PDI have also been found in different plants, such as *Oryza sativa*, *Triticum aestivum* L., *Brassica rapa* ssp. *pekinensis*, *Zea mays* and *Arabidopsis thaliana* [18–21]. *Arabidopsis* contains at least 12 PDI members, and they are divided into 3 groups based on polypeptide length, the presence of signal peptides and ER retention signals, and the composition of thioredoxin domains [19, 22–25]. *At*PDI1 (encoded by At3g54960) belongs to group II, having a typical PDI structure with 4 modular domains (*a, b, b′, a′*) and a CXXC motif in each *a* and *a′* domain, respectively [22]. In contrast to *h*PDI, an additional signal peptide is found in the amino terminus that targets chloroplasts, although *At*PDI1 also contains the ER retention signal (KDEL sequence) at the carboxyl terminus [25]. Experiments showed that *At*PDI1 was distributed in both organelles [23, 24], suggesting that it may play roles in both the ER and chloroplasts.

As one of the most important organelles, chloroplasts contain a considerable proportion of proteins whose activities can be changed by thiol-disulfide exchange [24, 26]. In *Arabidopsis* chloroplast stroma, *m*-type thioredoxin (Trx *m*) is the most abundant type of protein and it mainly catalyzes the reduction of disulfide bonds involved in photosynthetic carbon assimilation and accumulation in photosystem II [26], while the roles of oxidation or the formation of disulfide bonds remain uncertain. *At*PDI1 is most likely to oxidize the reduced thiol group in chloroplast stroma, catalyzing the exchange of thiol-disulfide [24]. In the ER, similar to animal PDI, *At*PDI1 is also the key enzyme catalyzing oxidative folding of secretory proteins and is also related to ER stress during the UPR [22].

Since plants are immobile, they are easily subjected to abiotic stresses. Similar to biotic stresses in humans (pathogen infection), abiotic stresses also lead to unfolded and misfolded proteins, causing loss of functional activities [27]. Recent studies have shown that some plant PDI members are involved in abiotic stresses [28–32]. Among 12 *Arabidopsis* PDIs, *At*PDI1 is strongly induced by abiotic stresses and exogenous abscisic acid (ABA) [28, 33, 34]. Our previous study showed that the *pdi* (*At*PDI1 T-DNA insertion knockdown mutant line) was more sensitive to abiotic stresses than WT *Arabidopsis*. Expression of recombinant *At*PDI1 significantly improved the resistance to abiotic stresses in *E. coli* [28]. To clarify whether the anti-stress ability of *At*PDI1 is related to its catalytic activity, here, the conserved cysteines in the CXXC motif(s) were mutated, and the *pdi* mutant line was used as the transgenic plant host. Two double mutants, *AtPDI1*_*m1*_ (*At*PDI1_C128AC131A_) and *AtPDI1*_*m2*_ (*At*PDI1_C467AC470A_), and wild-type *AtPDI1* (*At*PDI1), were constructed with the plasmids and successfully transformed into the *pdi* line. The transgenic lines were named *pdi*-*At*PDI1_m1_, *pdi*-*At*PDI1_m2_ and *pdi*-*At*PDI1. Then, the above lines, together with *pdi*, the WT and an overexpression line of the WT (WT-*At*PDI1), were all treated with different stresses to study whether the thiol-disulfide exchange activity of *At*PDI1 is involved in the response to abiotic stresses.

## Results

### In contrast to wild-type *AtPDI1*, mutations in *AtPDI1* could not compensate for the phenotype of *pdi* under abiotic stresses during germination

**Fig. 1:**
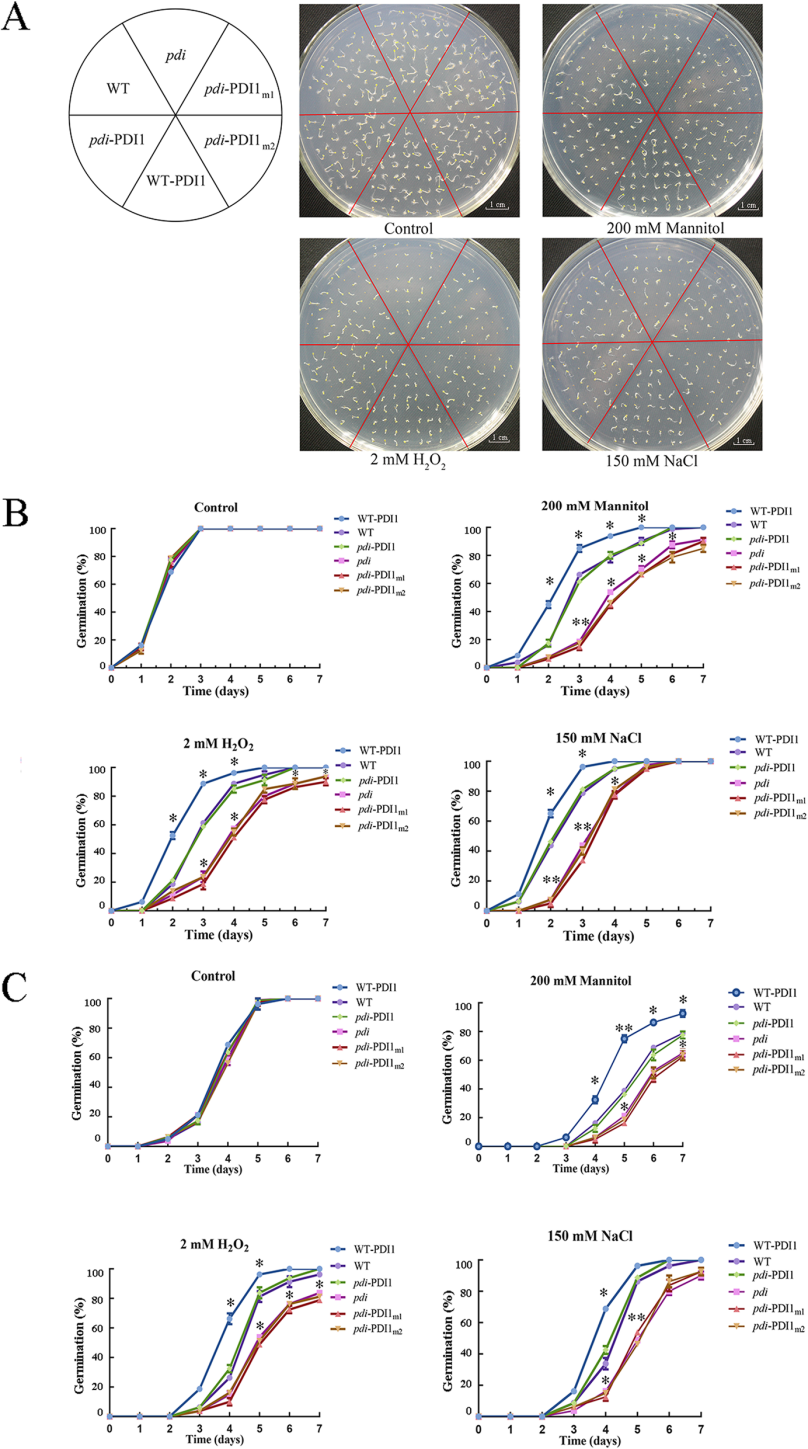
Seed germination of different *At*PDI1 lines on 1/2 MS medium containing H_2_O_2_, NaCl or mannitol. **A** Phenotypes of different *At*PDI1 lines (WT, *pdi*, WT-*At*PDI1, *pdi*-*At*PDI1, *pdi*-*At*PDI1_m1_ and *pdi*-*At*PDI1_m2_) under abiotic stress. **B** Gemination process according to radicle germination. **C** Gemination process according to cotyledon germination. Each experiment was carried out in three biological replicates

The seeds of different lines, including those of *pdi*, WT, WT-*At*PDI1, *pdi-At*PDI1, *pdi-At*PDI1_m1_ and *pdi-At*PDI1_m2,_ were all sown on the same stress medium to check whether the conserved cysteine residues have critical roles in the response to abiotic stresses. Two statistical methods, radicle appearance and cotyledon extension, were used to investigate the process of germination. Under normal conditions (1/2 MS medium), no difference in germination rate or germination speed was observed among the 6 lines when the radicle appearance was taken as the criterion, with germination reaching 100% at approximately 3 d (Fig. 1A, B). However, when the seeds were sown on medium containing 150 mM NaCl, 200 mM mannitol or 2 mM H_2_O_2_, significant differences in germination were observed (Fig. 1A, B). Under 200 mM mannitol treatment, the *pdi* line germinated much slower than the WT line, with an average of 15% vs. 65% germination in 3 d, respectively, whereas the WT *AtPDI1* successfully competed for the deficient phenotype of the *pdi* line; that is, the germination rate of *pdi*-*At*PDI1 was similar to that of the WT, reaching approximately 65% germination in 3 d. Most notably, the germination of *pdi-At*PDI1_m1_ and *pdi-At*PDI1_m2_ was quite similar to that of *pdi*, suggesting that the mutations of the conserved cysteines in the CXXC motif caused *At*PDI1 to completely lose its antistress ability during germination. Overexpression of *AtPDI1* enhanced the anti-stress ability and increased the germination of WT-*At*PDI1 compared with that of the WT (Fig. 1B). In addition, a similar tendency occurred with the 2 mM H_2_O_2_ and 150 mM NaCl treatments_._ When monitoring cotyledon extension during germination under different stresses, a similar tendency among different lines was observed (Fig. 1C).

**Fig. 2:**
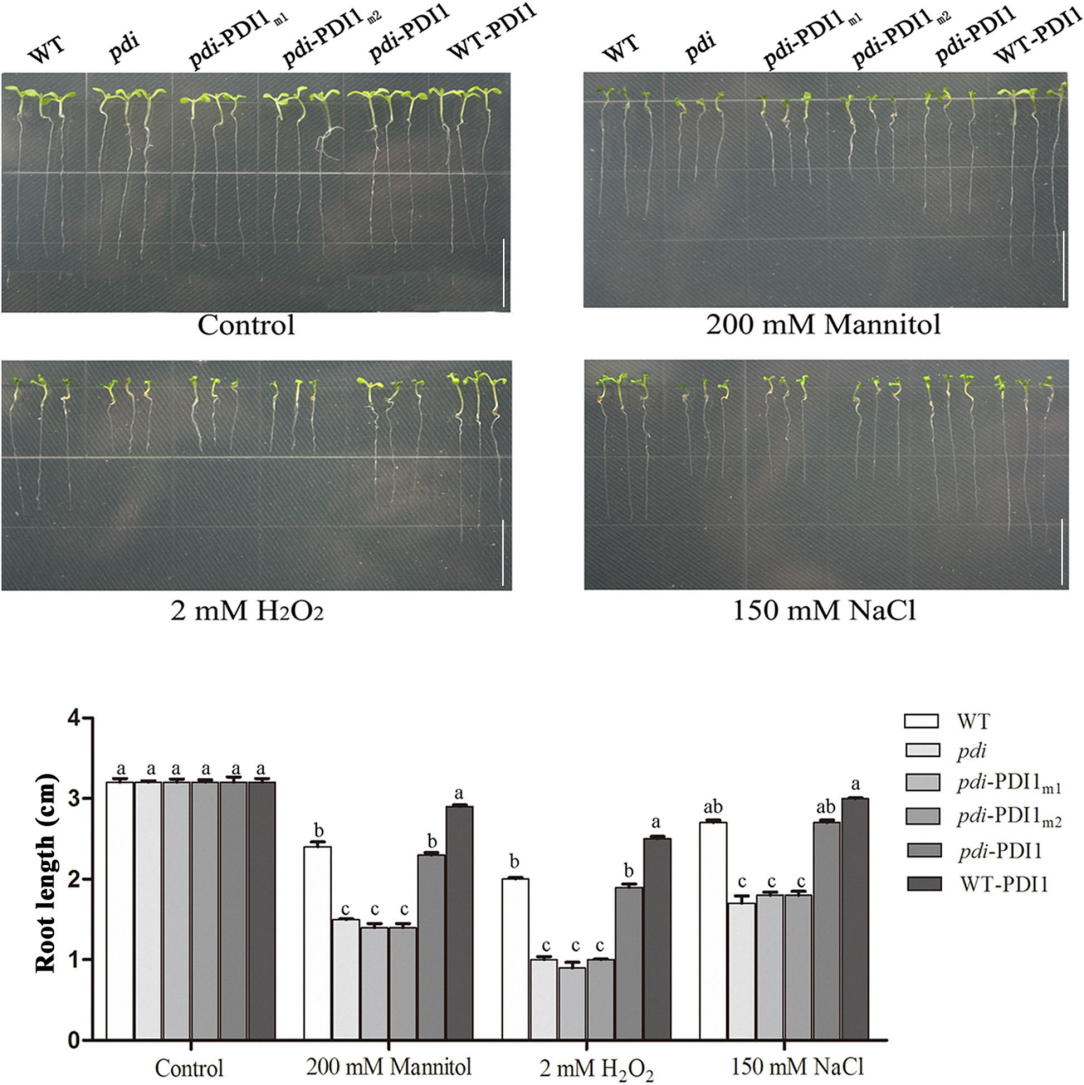
Root length of WT, *pdi*, WT-*At*PDI1, *pdi*-*At*PDI1, *pdi*-*At*PDI1_m1_ and *pdi*-*At*PDI1_m2_ lines on medium containing H_2_O_2_, NaCl or mannitol for 10 days, and the control for 7 days. The statistical significance level is shown using different letters. Each experiment was carried out in three biological replicates

To assay the difference in root length among the 6 lines, the seeds were sown on 1/2 MS medium until radicle appearance and then transferred to longitudinal stress medium containing NaCl, mannitol or H_2_O_2._ As shown in Fig. 2, although little difference was found among the 6 lines under normal conditions, significant differences in root length were observed among them under the abiotic stress treatments. The WT and *pdi*-*At*PDI1 lines had almost the same root length, and overexpression of *AtPDI1* (WT-*At*PDI1) promoted root length, while the root length of the other transgenic lines (*pdi*, *pdi-At*PDI1_m1_, *pdi-At*PDI1_m2_) was significantly shorter. The results collectively indicated that the mutations of the cysteines in *At*PDI1 inhibited root growth under abiotic stresses (Fig. 2).

### Mutations of the active site in *AtPDI1* decreased seedling resistance to abiotic stresses

**Fig. 3:**
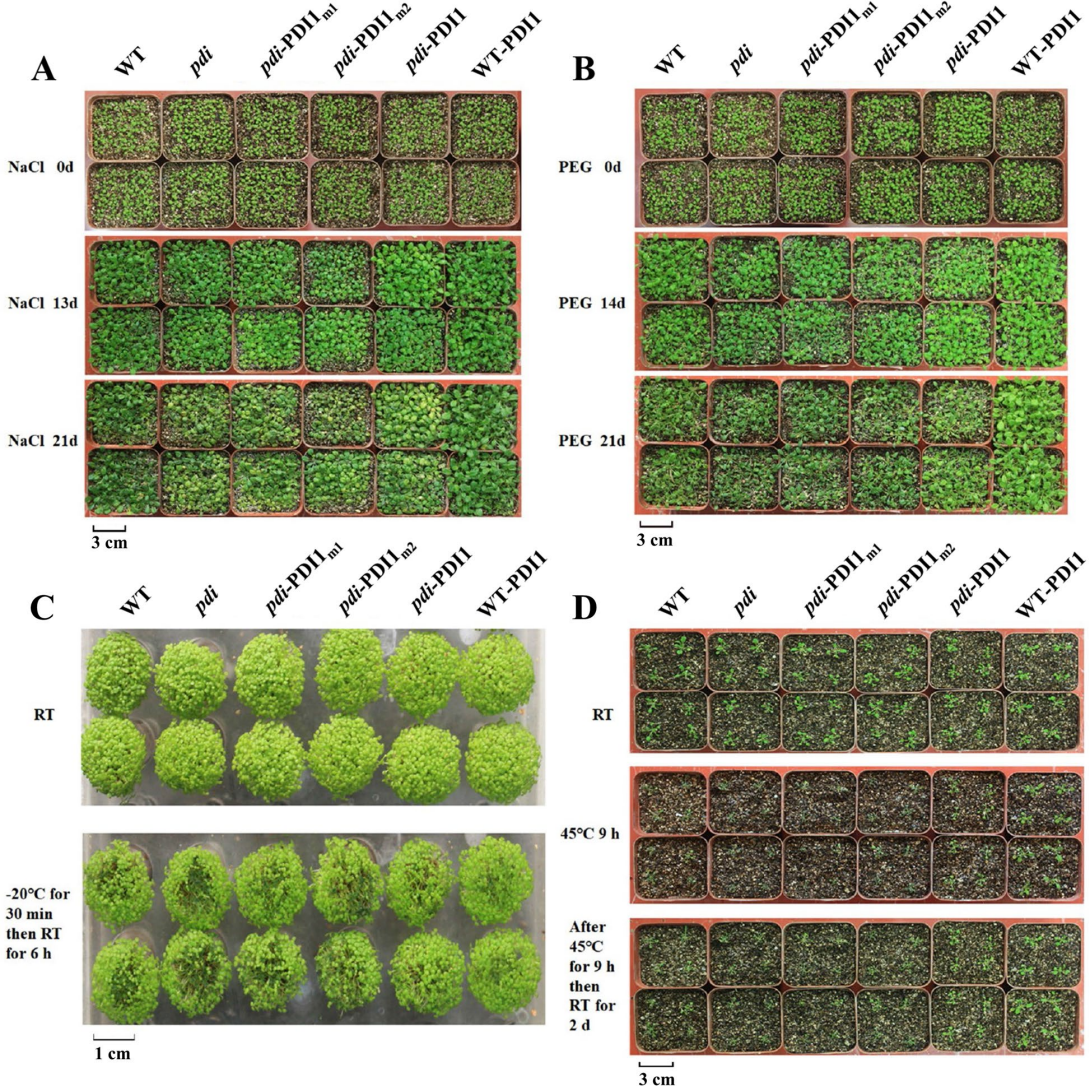
Phenotypes of WT, *pdi*, WT-*At*PDI1, *pdi*-*At*PDI1, *pdi*-*At*PDI1_m1_ and *pdi*-*At*PDI1_m2_ lines under different abiotic stresses. **A** 250 mM NaCl. **B** 30% (w/v) PEG6000. **C** − 20 °C for 30 min and then RT for 6 h. **D** 45 °C for 9 h and then RT for 2 d

Seedlings of different lines of the same age were treated with salt, osmotic and extreme temperature stresses. When 7-day-old seedlings were watered with 250 mM NaCl for 13 d, little difference among different lines was observed. However, when the treatment lasted for 21 d, significant differences emerged. The WT and *pdi*-*At*PDI1 lines grew well and had a similar etiolation rate (approximately 35%), while the *pdi*, *pdi-At*PDI1_m1_ and *pdi-At*PDI1_m2_ lines turned yellow or even died, with an etiolation rate of approximately 70%, the WT-*At*PDI1 plants grew best with an etiolation rate of only 8% (Fig. 3A, Fig. S1A). After PEG treatment for 21 d, the WT-*At*PDI1 lines displayed a higher survival rate (80%) than the other lines, whereas the survival rate of the *pdi* (52%) line was the lowest, followed by the *pdi*-*At*PDI1_m1_ (62%) and *pdi*-*At*PDI1_m2_ lines (68%) (Fig. S1B).

To test the effects of *AtPDI1* on extreme temperature, seedlings were placed in a low- or high-temperature environment. For cold treatment, the seedlings were placed at − 20 °C for 30 min and then placed at room temperature (RT) for 6 h. As shown in Fig. 3C, compared with *pdi*-*At*PDI1 or WT plants, the plants of the *pdi*, *pdi-At*PDI1_m1_ and *pdi-At*PDI1_m2_ lines showed weak tolerance to cold stress, many seedlings exhibited damage and increased leaf juice effusion, with a survival rate of only 63–67% (Fig. S1C), whereas the overexpression line (WT-*At*PDI1) could better withstand cold stress, with a survival rate of 90.3% (Fig. S1C). For the high-temperature treatment, seedlings were treated at 45 °C for 9 h and then placed under normal conditions. After 2 days at RT, WT-*At*PDI1 displayed the highest survival rate (100%), the WT fared better than *pdi-At*PDI1, with a survival rate of 90%, while most plants of the *pdi*, *pdi-At*PDI1_m1_ and *pdi-At*PDI1_m2_ lines still remained in a withered stated, with a survival rate of approximately 58–75% (Fig. 3D, Fig. S1D). These results suggested that the conserved cysteines in the active site of *At*PDI1 played critical roles in resisting abiotic stresses.

### Activities of thiol-disulfide exchange of *At*PDI1 affect ABA sensitivity during seed germination

**Fig. 4:**
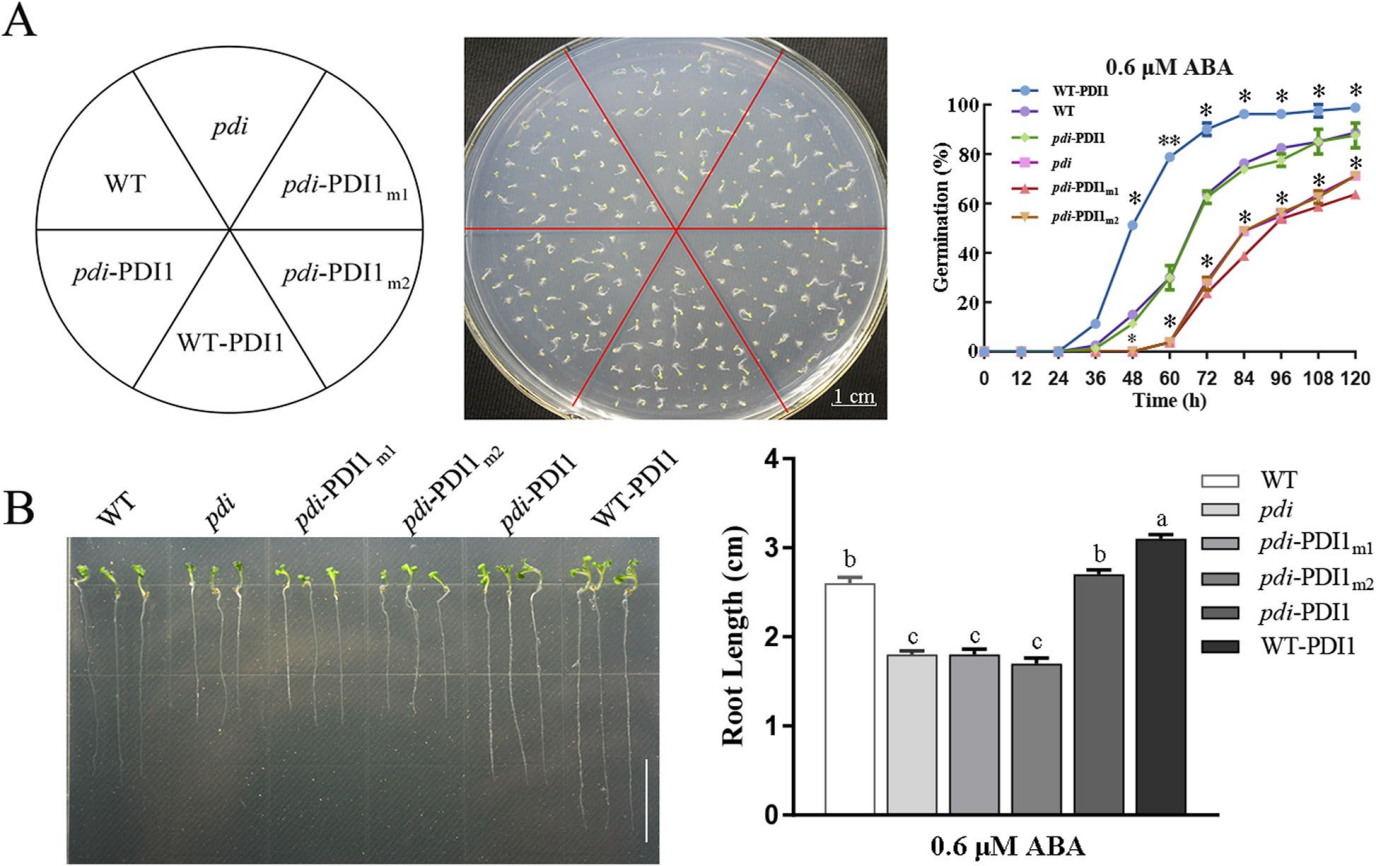
Seed germination and growth of WT, *pdi*, WT-*At*PDI1, *pdi*-*At*PDI1, *pdi*-*At*PDI1_m1_ and *pdi*-*At*PDI1_m2_ lines under ABA treatment. **A** The rates of seed germination of different *At*PDI1 lines under 0.6 μM ABA during the germination process. **B** The root lengths of *At*PDI1 lines under 0.6 μM ABA treatment for 14 days

ABA signaling is involved in seed germination. To verify whether the change in *At*PDI1 activities affects the plant tolerance to ABA, different lines were treated with exogenous ABA to observe the seed germination rate and root length. The results showed that the WT-*At*PDI1 line exhibited the highest germination rates and the longest roots among the 6 lines, suggesting that overexpression of *AtPDI1* decreased ABA sensitivity. The WT and *pdi-At*PDI1 lines had a similar germination rate, which was lower than that of WT-*At*PDI1 but higher than that of *pdi*, *pdi-At*PDI1_m1_ and *pdi-At*PDI1_m2_, and the germination rates of the latter three lines showed no obvious differences. In addition, the difference in root length among these 6 lines coincided with the germination rates (Fig. 4). In addition, the difference in root length among these 6 lines coincided with the germination rates (Fig. 4). The above results indicated that the change in *At*PDI1 activities affects the tolerance to ABA.

### *At*PDI1 functions in the salt stress response may be related to the ABA signaling pathway

**Fig. 5:**
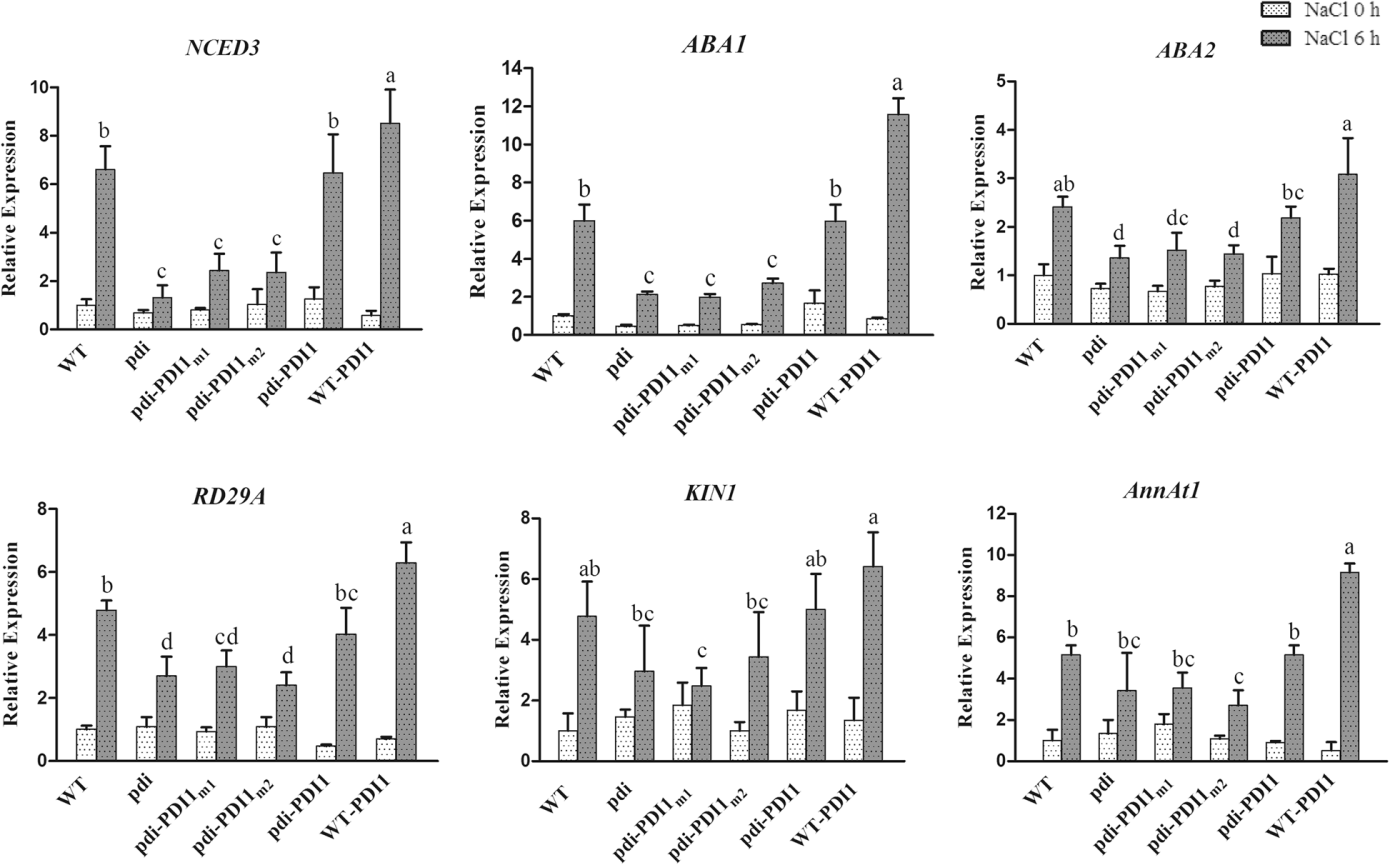
The relative mRNA levels of genes related to the ABA pathway were detected in *At*PDI1 lines (WT, *pdi*, WT-*At*PDI1, *pdi*-*At*PDI1, *pdi*-*At*PDI1_m1_ and *pdi*-*At*PDI1_m2_) under NaCl stress treatment. The results are presented as the mean values of three independent replicates. Each reaction was carried out in three biological replicates. The statistical significance level is shown using different letters. (Student’s t test; *P* < 0.05)

To further investigate the relationship between the changes in the stress resistance of the mutant *At*PDI1 and the ABA signaling pathway, the expression of key genes related to ABA synthesis (*ABA1, ABA2, NCED3*) and ABA response (*RD29A, KIN1, AnnAt1*) was assayed. As shown in Fig. 5, all the above genes were upregulated under NaCl treatment; however, the range of increase was different. The ranges of the expression changes in the abovementioned genes in *pdi*, *pdi-At*PDI1_m1_ and *pdi-At*PDI1_m2_ were similar and were significantly less than those in the WT and *pdi-At*PDI1. For example, the expression of *ABA1* was upregulated 12-fold for WT-*At*PDI1, approximately 6-fold for WT and *pdi-At*PDI1, and less than 3-fold for *pdi*, *pdi-At*PDI1_m1_ and *pdi-At*PDI1_m2_. Taken together, the above results preliminarily suggested that *At*PDI1 might be involved in the ABA-dependent pathway to enhance resistance to abiotic stresses.

### Activities of thiol-disulfide exchange of *At*PDI1 are also related to ROS elimination

**Fig. 6:**
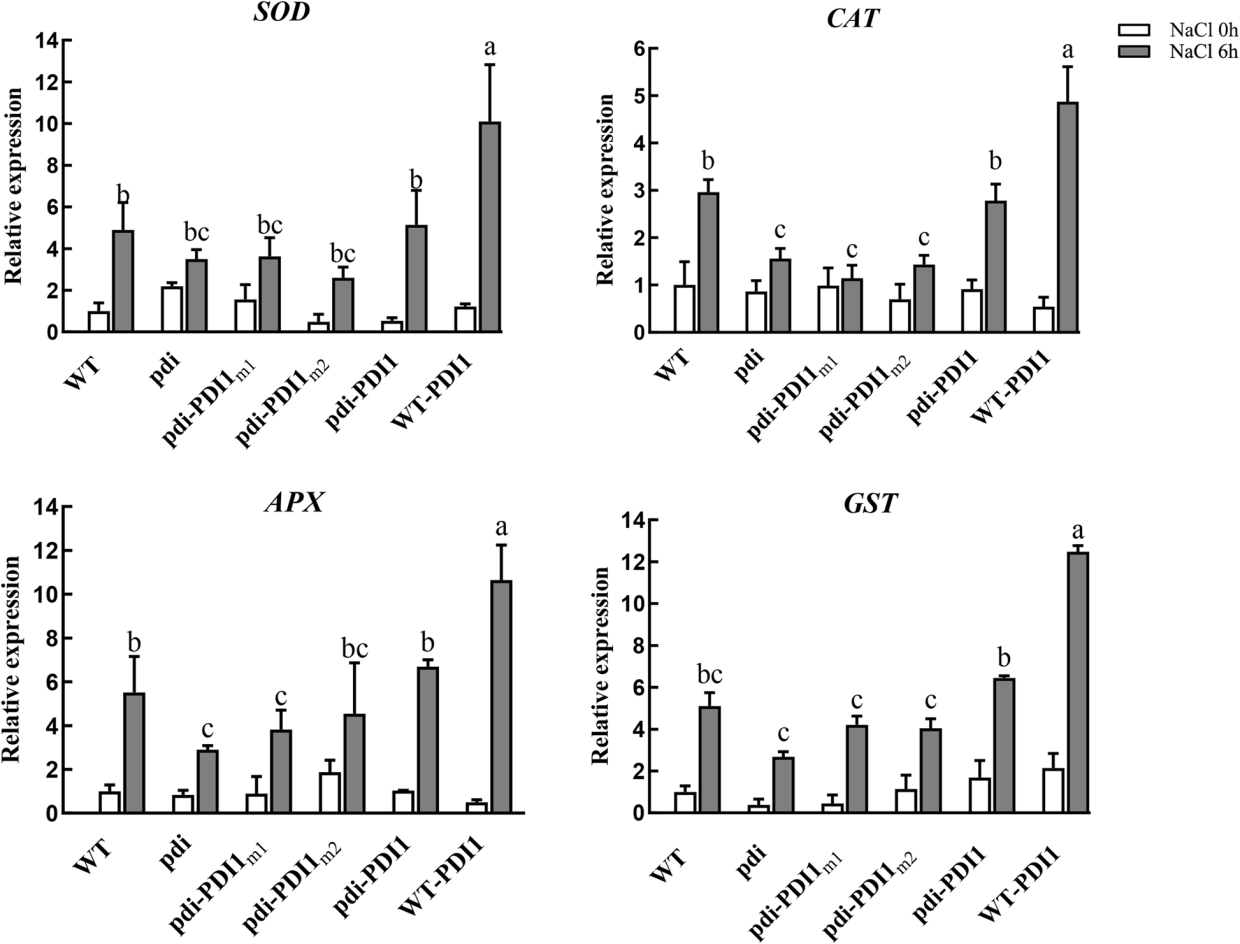
Relative expression levels of ROS quenching enzymes in different *At*PDI1 lines. The WT, *pdi*, WT-*At*PDI1, *pdi*-*At*PDI1, *pdi*-*At*PDI1_m1_ and *pdi*-*At*PDI1_m2_ plants were treated with 250 mM NaCl for 6 h. The results are presented as the mean values of three independent replicates. Each reaction was carried out in three biological replicates. The statistical significance level is shown using different letters. (Student’s t test; P < 0.05)

Reactive oxygen species (ROS) include H_2_O_2_, O_2_^·-^, OH^.^ and singlet oxygen [35, 36]. Abiotic stress could result in the production of ROS via the deregulation of electron transport in the chloroplast and mitochondria [35, 37]. ROS accumulation in chloroplasts can significantly inhibit the de novo synthesis of D1 protein, thus inactivating the PSII repair process [38]. In addition to causing toxicity, ROS can regulate plant development and act as signaling molecules of abiotic stress [35, 37]. Some enzymes, including superoxide dismutase (SOD), catalase (CAT), L-ascorbate peroxidase (APX) and glutathione transferase (GST), have ROS-quenching functions and maintain the balance of ROS in organisms. The transcriptional expression of these enzymes was detected by qRT-PCR. Compared with the lower levels of expression before the abiotic stresses were applied, the expression of these genes was upregulated after the seedlings were treated with 250 mM NaCl. The expression of the genes in the *pdi*, *pdi-At*PDI1_m1_ and *pdi-At*PDI1_m2_ plants was altered less than that in the WT and *pdi*-*At*PDI1 lines, while that in WT-*At*PDI1 was upregulated the most (Fig. 6).

**Fig. 7:**
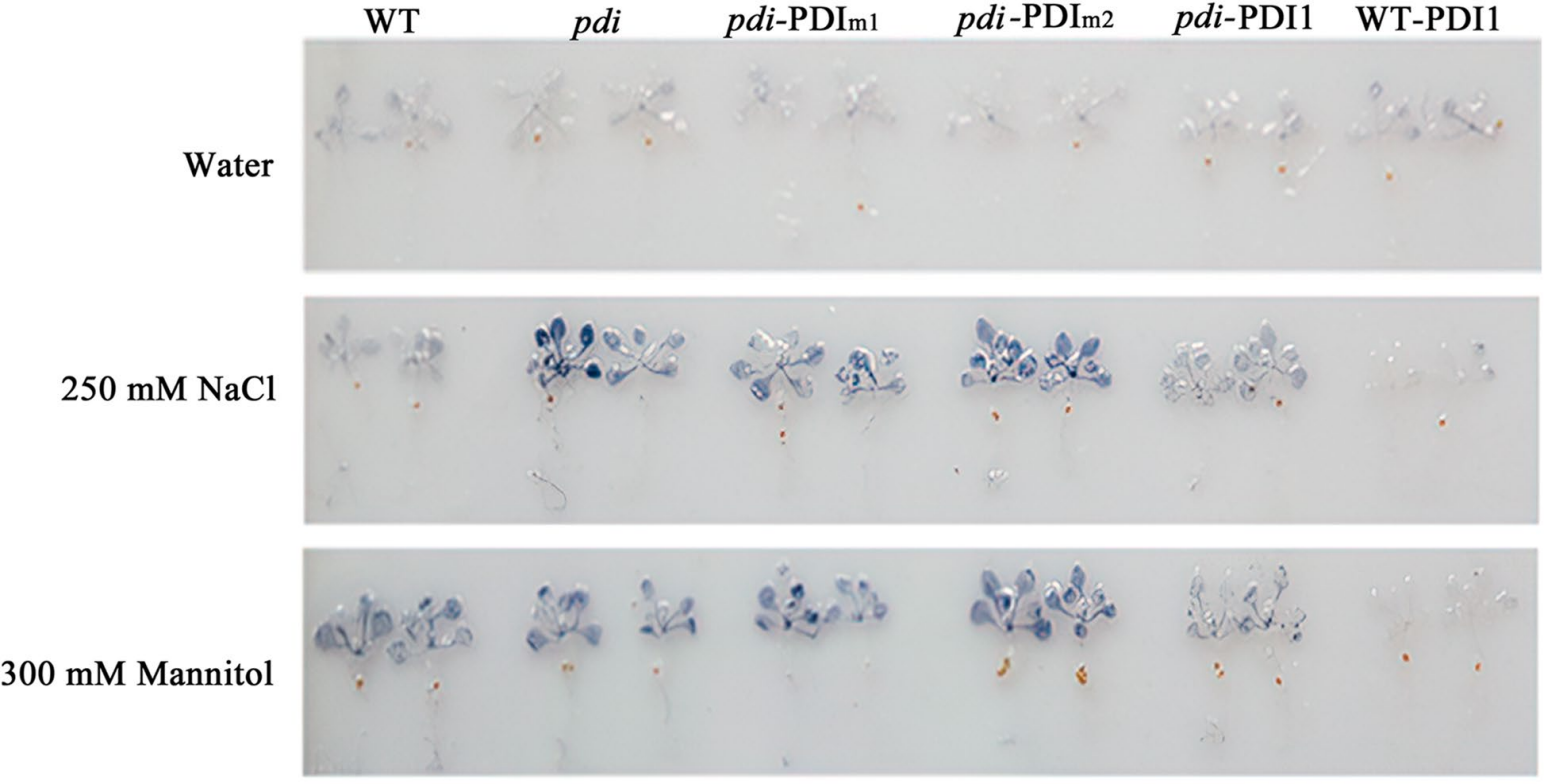
NBT staining of O_2_^**.**-^ of WT, *pdi*, WT-*At*PDI1, *pdi*-*At*PDI1, *pdi*-*At*PDI1_m1_ and *pdi*-*At*PDI1_m2_ lines under treatment with 250 mM NaCl and 300 mM mannitol. Water was applied to the control

In addition, NBT staining was used to measure the accumulation of O_2_^·-^ in different lines under 250 mM NaCl and 300 mM mannitol treatments. More O_2_^.-^ accumulated in *pdi*, *pdi-At*PDI1_m1_ and *pdi-At*PDI1_m2_ seedlings with deeper staining than in WT, *pdi-At*PDI1 and OE plants (Fig. 7). The accumulation of H_2_O_2_ was also checked by DAB staining, but no significant differences among different lines were observed.

## Discussion

When the human body is threatened by diseases and other adverse environments, folding-incompetent proteins accumulate, and then ER stress and an associated stress response, called the unfolded protein response (UPR), appear [39]. As important members of the protein folding machinery in the ER, extensive studies have shown that PDIs can catalyze the formation and rearrangement of disulfide bonds of proteins and play a critical role in nascent peptide folding [39–41]. *h*PDI promotes protein folding by catalyzing the exchange of thiol-difulfide, which is dependent on the CXXC motif in the *a* and *a′* domains [42].

In plants, PDI is also involved in defense against abiotic stresses, which often disturb cell physiological processes and lead functional proteins to unfold or misfold [20, 29]. Similar to *h*PDI, *At*PDI1 has a typical modeling structure of PDI, and some *Arabidopsis* PDIs are induced to improve protein folding and transport, which subsequently regulate the protein networks under stresses [33]. In this study, we found that the anti-stress activity of *At*PDI1 was related to its catalytic activity domain. The *pdi* line was the most sensitive among the 6 lines under different abiotic stress treatments; however, when *At*PDI1 was supplemented in the *pdi* line (*pdi*-*At*PDI1), the deficient phenotype was restored, and the growth state was consistent with that of the WT. Although *pdi*-*At*PDI1_m1_ and *pdi*-*At*PDI1_m2_ were successfully expressed in the *pdi* line (Fig. S1), this could not compensate for the deficiency of *pdi*, and these lines were still sensitive to stresses, similar to the *pdi* line. The results suggested that the antistress roles of *At*PDI1 are directly related to the activity of thiol-disulfide exchange.

The ABA signaling pathway is an important regulatory mechanism in response to abiotic stress in plants, including salinity, drought and cold [28]. A previous study revealed that the transcript level of *AtPDI1* was induced by abiotic stresses and exogenous ABA [28], which suggested that the anti-stress function of *AtPDI1* may be related to ABA signaling pathway. In this study, the results showed that the activity of thiol-disulfide exchange affected the resistance to abiotic stress. Moreover, the transcription levels of ABA-responsive genes were substantially higher in WT-*At*PDI1 than in WT and the *AtPDI1* mutant lines under salt stress conditions, suggesting that *AtPDI1* activity is involved in abiotic stress resistance by regulating the expression of ABA-responsive genes.

The change in the activities of *At*PDI1 also affected seed sensitivity to ABA, and mutation of *At*PDI1 increased sensitivity to ABA, with lower germination and shorter root length under exogenous ABA treatment. Many investigations reported that some genes overexpression exhibited ABA hypersensitive phenotypes during seed germination and improved abiotic stress tolerance through ABA-dependent pathway [42]. However, overexpression of some genes displayed different effects, with ABA -insensitive phenotypes during seed germination and increased stress tolerance through ABA-dependent pathway. These genes come from different plants, such as *GhMYB73* [43], *TaMYB73* [44], *OsMYB3R-2* [45], and *AtHD2C* [46]. Our results showed that *Arabidopsis* overexpressing *AtPDI1* had a similar phenotype to *Arabidopsis* overexpressing the above genes, affecting abiotic stress resistance through ABA signaling pathway. However, how *AtPDI1* is involved in the ABA pathway to increase abiotic tolerance needs further study.

Abiotic stress can result in the accumulation of excessive ROS, which have been proven to have a negative effect on abiotic stress resistance in plants. Therefore, an increased ROS-scavenging ability might be beneficial to plant tolerance to abiotic stresses [37]. Conservative cysteine mutations of PDI also affected the activities of antioxidant enzymes and ROS production [47, 48]. In this study, the expression levels of ROS scavenging enzyme genes in mutant plants were also much lower than those in WT and OE lines. In addition, the amount of ROS was higher in mutant *At*PDI1 plants with conserved cysteines. These results suggested that the catalytic activities of *At*PDI1 may be involved in the ROS pathway by regulating the activities of some proteins, but this remains to be further studied.

## Conclusion

Based on our findings and previous reports, a hypothetical scheme (Fig. S5) was proposed to depict the molecular basis of *AtPDI1* involved in the abiotic stress response. Abiotic stress can cause the accumulation of unfolded and misfolded proteins whose disulphide bonds are disordered and disorganized. *At*PDI1 has disulfide isomerase and molecular chaperone activities, which can help these proteins fold correctly. Overexpression of *AtPDI1* in *Arabidopsis* significantly enhanced abiotic stress tolerance. However, mutations of conserved cysteines in the CXXC motif of *At*PDI1 decreased the anti-stresses ability of plants, which indicated that the anti-stresses function of *At*PDI1 is directly related to the activity of disulfide isomerase.

## Materials and methods

### Construction of plasmids and transformation into *Arabidopsis*

The open reading frame (ORF) of cysteine-mutant *AtPDI1* was amplified by PCR using plasmids previously constructed in our laboratory [28]. Two double mutants (*AtPDI1*_*m1*_ and *AtPDI1m*_*2*_) of conserved cysteines were amplified using a QuickChange® II Site-Directed Mutagenesis Kit (Agilent Technologies Inc., US), which changed the cysteines at the motif of Cys128XXCys131 and Cys467XXCys470 of *AtPDI1* to Tyr128XXTyr131 and Tyr467XX Tyr470, respectively. Then, all the sequences were inserted into the plasmid of pROKII-GFP containing the CaMV35S promoter by T4 ligase (Fig. S2). The primers used in this investigation are listed in Supplementary Table S1. All recombinant plasmids were confirmed by Shanghai Sunny Biotechnology Co. (Shanghai, China), and the recombinant vectors were transformed into *Agrobacterium* GV3101 using floral dip transformation. Transgenic plants were selected on the basis of kanamycin resistance and verified by genomic PCR with specific primers (Fig. S3). The homozygous lines were obtained from the T3 generation (Fig. S4).

### Plant materials and growth condition

The wild-type *Arabidopsis thaliana* used in this study was the Columbia ecotype (Col-0) provided by Prof. Chengchao Zheng (Shandong Agricultural University, China), which was originally obtained from Arabidopsis Biological Resource Center (ABRC) [28], and overexpression lines (WT-*At*PDI1) were obtained by Zhengrong Zhang as described in our previous study [28]. An *AtPDI1* T-DNA insertion knockdown mutant line, *pdi* (Salk_150463C), was obtained from ABRC and verified by Zhengrong Zhang [28]. Complementary lines (*pdi*-*At*PDI1, *pdi*-*At*PDI1_m1_ and *pdi*-*At*PDI1_m2_) were obtained and identified by Li Yuan in this study as described in the last section, which has not been deposited in a publicly available herbarium. First, the relative mRNA levels of *AtPDI1* in the above lines were examined under normal conditions (Fig. S4). Then, the seeds of WT, *pdi* and transgenic plants were sown on Murashige and Skoog (MS) agar medium in greenhouse conditions at 22 °C under short sunshine conditions with an 8 h light/16 h dark cycle and then transplanted into vermiculite of Shandong Agricultural University under the conditions described by a previous study [28].

Seed germination and root length experiments were performed as described by Zhang et al. (2018) [28]. The washed seeds were germinated on 1/2 MS agar medium with 0.6 mM ABA, 150 mM NaCl, 200 mM mannitol, or 2 mM H_2_O_2_ added. Each experiment was carried out in three biological replicates.

For salt or drought stress treatment, 7-day-old wild-type and transgenic plants were irrigated with 250 mM NaCl or 30% (w/v) polyethylene glycol-6000 (PEG-6000) instead of water, respectively. The growth status and the survival rates of 50 seedlings were observed and recorded during the following 21 days. For extreme temperature, 7-day-old wild-type and transgenic plants were treated at − 20 °C for 30 min and then held at room temperature (RT) for 6 h or at 45 °C for 9 h and then subjected to RT for 2 d. Each experiment was carried out in three biological replicates.

### RNA extraction and quantitative real-time PCR (qRT-PCR) analysis

Normally grown *Arabidopsis* plants were treated with solutions containing 150 mM NaCl or with water as a control. The leaves were collected at 6 h, and the total RNA was extracted from the material. Qualified RNA was used to synthesize cDNA with a cDNA synthesis kit (Vazyme, Nanjing, China). The *GAPDH* gene from *Arabidopsis* was used as an internal control for expression normalization in qRT-PCR as done in similar investigations, and the relative expression levels were calculated by the 2^− △  △ Ct^ method [49]. The specific primers used for qRT-PCR are listed in Table S1. The expression levels of ABA- and ROS-related genes in stressed samples (6 h) were compared to the control levels with Student’s t test at a significance level of *P* < 0.05. Each reaction was carried out in three biological replicates.

### ROS determination

Normally grown 3-week-old *Arabidopsis* seedlings were treated with solutions containing 250 mM NaCl or 300 mM mannitol, and water was applied at room temperature as a control. The contents of H_2_O_2_ and O_2_^·-^ were determined by nitroblue tetrazolium (NBT) staining according to a previous method [48]. Each experiment was carried out in three biological replicates.

## Supplementary Information

**Additional file 1: Table S1**. Primers used in this experiment.

**Additional file 2: Figure S1**. The etiolation rate or survival rate of WT, *pdi*, WT-*At*PDI1, *pdi*-*At*PDI1, *pdi*-*At*PDI1_m1_ and *pdi*-*At*PDI1_m2_ lines under different abiotic stresses. (A) The etiolation rate under 250 mM NaCl treatment. (B) The survival rate after 30% (w/v) PEG6000 treatment. (C) The survival rate after − 20 °C for 30 min and then RT for 6 h. (D) The survival rate after 45 °C for 9 h and then RT for 2 d. The statistical significance level is shown using different letters. (Student’s t test; *P* < 0.05).

**Additional file 3: Figure S2**. Diagram of the plasmid vector pROKII with *PDI1m1* and *PDI1m2.* NPTII, neomycin phosphotransferase II. CaMV 35S, cauliflower mosaic virus 35S promoter. NOS-ter, terminator of nitric oxide synthase. NOS-pro, promoter of nitric oxide synthase.

**Additional file 4: Figure S3**. Screening and PCR detection of transgenic seedlings by Kanamycin. A, B, C represent transgenic seeding of *pdi-PDI1*, *pdi-PDI1m1* and *pdi-PDI1m2*, respectively; D, E, F represent PCR products of *pdi-PDI1*, *pdi-PDI1m1* and *pdi-PDI1m2*, respectively.

**Additional file 5: Figure S4**. Screening and qRT-PCR analysis of homozygous transgenic plants. A, B, C represent *pdi-PDI1*, *pdi-PDI1m1* and *pdi-PDI1m2*, respectively, and the middle yellow seedling in every plate is the *pdi* mutant. D represents the expression levels of *AtPDI1* in different lines (WT, *pdi*, WT-*At*PDI1, *pdi*-*At*PDI1, *pdi*-*At*PDI1_m1_ and *pdi*-*At*PDI1_m2_) under normal conditions. The results are represented as the mean values of three independent replicates. Each reaction was carried out in three biological replicates. The statistical significance level is shown using different letters. (Student’s t test; *P* < 0.05).

**Additional file 6: Figure S5**. A hypothetical scheme depicting the molecular basis of AtPDI1 involved in the abiotic stress response.

## Data Availability

The datasets used and/or analyzed during the current study are available from the corresponding author on reasonable request.

## Abbreviations

PDI: Protein disulfide isomerase
ER: Endoplasmic reticulum
ABA: Exogenous abscisic acid
qRT-PCR: Quantitative realtime PCR
WT: Wild-type of Arabidopsis
ROS: Reactive oxygen species
SOD: Superoxide dismutase
CAT: Catalase
APX: L-ascorbate peroxidase
GST: Glutathione transferase
