# Supplementary material for: The thiol-disulfide exchange activity of AtPDI1 is involved in the response to abiotic stresses
Source: BMC Plant Biol. 2021 Nov 23;21:557. doi: 10.1186/s12870-021-03325-7 (PMC8609882; doi:10.1186/s12870-021-03325-7)
Supplement: Supplementary file 1 — Additional file 1: Table S1. Primers used in this experiment. [file 12870_2021_3325_MOESM1_ESM.docx]

Table S1 Primers used in this investigation

| Primer designations | Primer Sequences（5′-3′） |
| --- | --- |
| *At*PDI*1-pROKII-F* | GCTCTAGAATGGCTTCGTCATCTACAAG |
| *At*PDI*1-pROKII-R* | CGGGATCCCAACTCATCCTTGGAAC |
| *mAtPDI1_C128/131A_-F* | TTACGCTCCGTGGTACGGCGCTTATCAGGCTTTGAC |
| *mAtPDI1_C128/131A_-R* | TAAGCGCCGTACCACGGAGCGTAAAACTCCACCATC |
| *mAtPDI1_C467/470A_-F* | ATATGCTCCTTGGTATGGCCACTACCAATCATTTGA |
| *mAtPDI1_C467/470A_-R* | TAGTGGCCATACCAAGGAGCATATATCTCGAGAAGA |
| *GFP-F* | GCTCTAGAATGGCTTCGTCATCTACAAG |
| *GFP-R* | CGGGATCCCAACTCATCCTTGGAAC |
| *qAtGAPDH-F* | TGGTTGATCTCGTTGTGCAGGTCTC |
| *qAtGAPDH-R* | GTCAGCCAAGTCAACAACTCTCTC |
| *qAtABA1-F* | GACTGGGTCCTTGGAGGTAA |
| *qAtABA1-R* | CATCGGCTTTGTCAGTGAGT |
| *qAtABA2-F* | TCCAAGCATGCTGTTCTAGG |
| *qAtABA2--R* | AAATGAGCCAAAGCGAGTTT |
| *qAtNCED3-F* | TTGATGCTCCAGATTGCTTC |
| *qAtNCED3-R* | GGACCCTATCACGACGACTT |
| *qAtRD29A-F* | CTTGATGGTCAACGGAAGGT |
| *qAtRD29A-R* | CAATCTCCGGTACTCCTCCA |
| *qAtKIN1-F* | GCAATGTTCTGCTGGACAAG |
| *qAtKIN1-R* | TACACTCTTTCCCGCCTGTT |
| *qAtAnnAt1-F* | TTCTCTTGTTACCTCATAC |
| *qAtAnnAt1-R* | CATCCTCATCATTGTAGT |
| *qAtAPX-F* | ATATGCTGACTTCTACCA |
| *qAtAPX-R* | GTCTACCAGGATGAAATG |
| *qAtSOD-F* | TTGTGTTGTGACGACAAGC |
| *qAtSOD-R* | ATCAATCTGCTCAAGAACACC |
| *qAtCAT-F* | TATGGAACAACAACTCCTCC |
| *qAtCAT-R* | TCTCTGAGTATCGGCATAGG |
