# Supplementary figures and images for: The thiol-disulfide exchange activity of AtPDI1 is involved in the response to abiotic stresses
Source: BMC Plant Biol. 2021 Nov 23;21:557. doi: 10.1186/s12870-021-03325-7 (PMC8609882; doi:10.1186/s12870-021-03325-7)

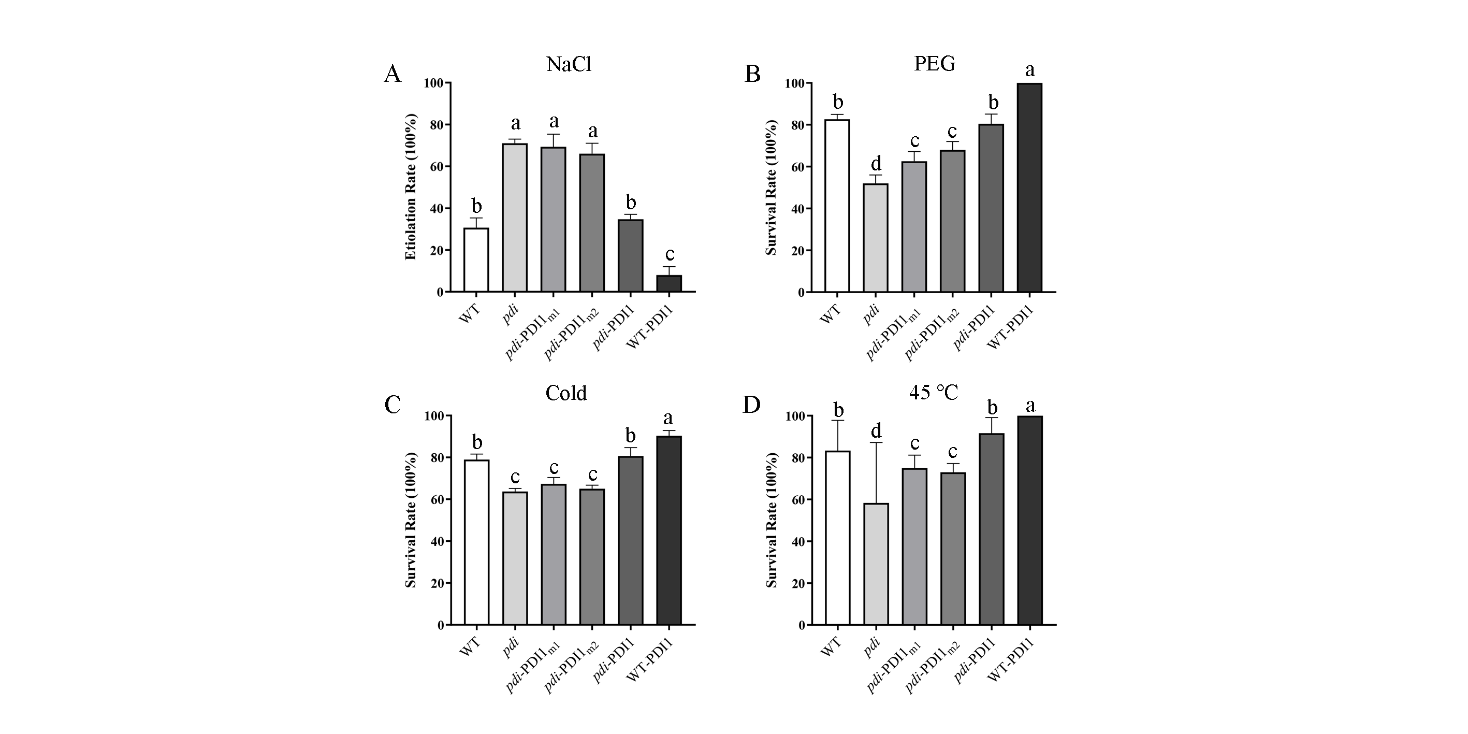

Supplement: Supplementary file 2 — Additional file 2: Figure S1. The etiolation rate or survival rate of WT, pdi, WT-AtPDI1, pdi-AtPDI1, pdi-AtPDI1m1 and pdi-AtPDI1m2 lines under different abiotic stresses. (A) The etiolation rate under 250 mM NaCl treatment. (B) The survival rate after 30% (w/v) PEG6000 treatment. (C) The survival rate after − 20 °C for 30 min and then RT for 6 h. (D) The survival rate after 45 °C for 9 h and then RT for 2 d. The statistical significance level is shown using different letters. (Student’s t test; P < 0.05). [file 12870_2021_3325_MOESM2_ESM.docx]

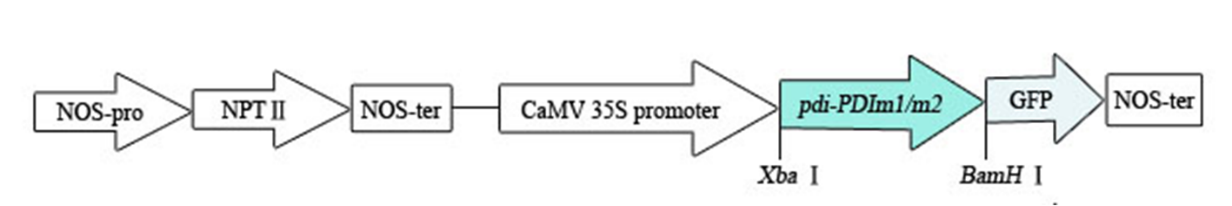

Supplement: Supplementary file 3 — Additional file 3: Figure S2. Diagram of the plasmid vector pROKII with PDI1m1 and PDI1m2. NPTII, neomycin phosphotransferase II. CaMV 35S, cauliflower mosaic virus 35S promoter. NOS-ter, terminator of nitric oxide synthase. NOS-pro, promoter of nitric oxide synthase. [file 12870_2021_3325_MOESM3_ESM.docx]

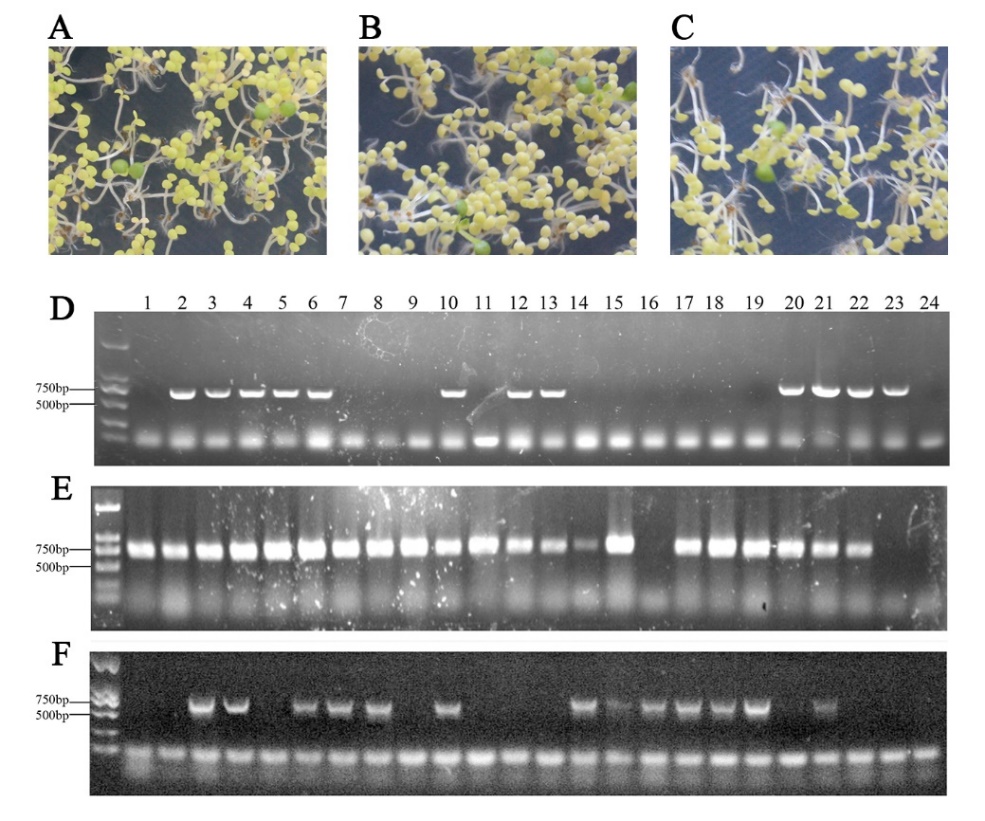

Supplement: Supplementary file 4 — Additional file 4: Figure S3. Screening and PCR detection of transgenic seedlings by Kanamycin. A, B, C represent transgenic seeding of pdi-PDI1, pdi-PDI1m1 and pdi-PDI1m2, respectively; D, E, F represent PCR products of pdi-PDI1, pdi-PDI1m1 and pdi-PDI1m2, respectively. [file 12870_2021_3325_MOESM4_ESM.docx]

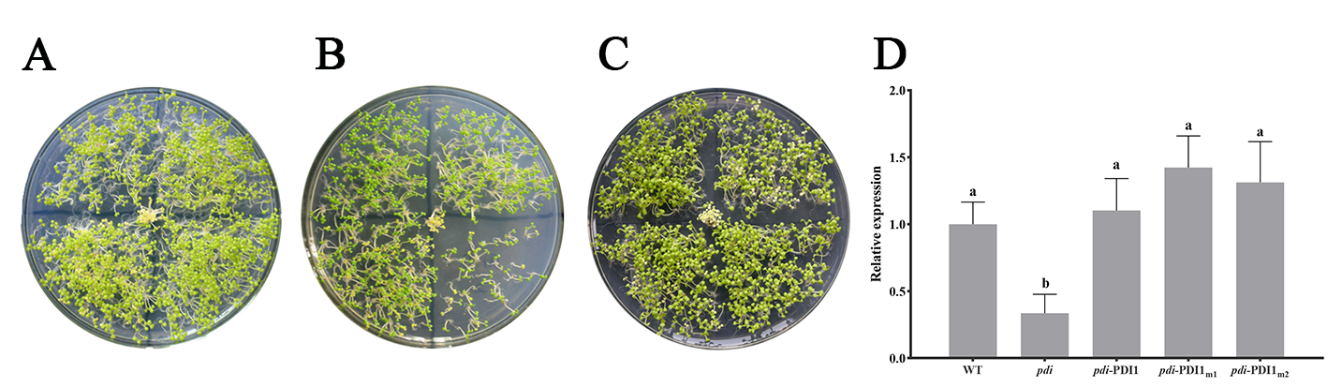

Supplement: Supplementary file 5 — Additional file 5: Figure S4. Screening and qRT-PCR analysis of homozygous transgenic plants. A, B, C represent pdi-PDI1, pdi-PDI1m1 and pdi-PDI1m2, respectively, and the middle yellow seedling in every plate is the pdi mutant. D represents the expression levels of AtPDI1 in different lines (WT, pdi, WT-AtPDI1, pdi-AtPDI1, pdi-AtPDI1m1 and pdi-AtPDI1m2) under normal conditions. The results are represented as the mean values of three independent replicates. Each reaction was carried out in three biological replicates. The statistical significance level is shown using different letters. (Student’s t test; P < 0.05). [file 12870_2021_3325_MOESM5_ESM.docx]

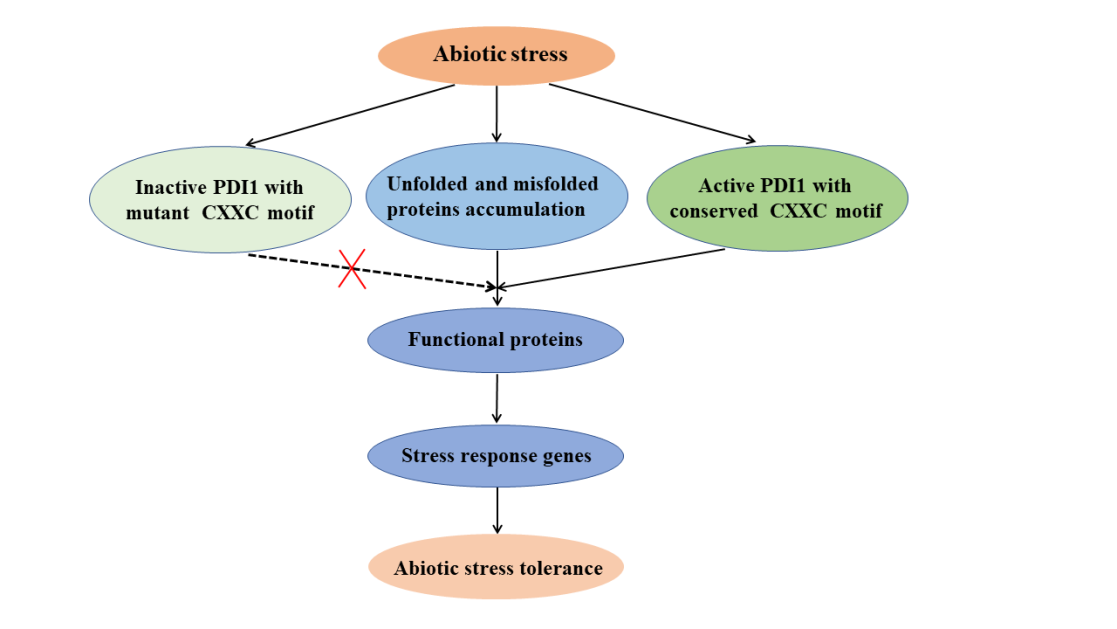

Supplement: Supplementary file 6 — Additional file 6: Figure S5. A hypothetical scheme depicting the molecular basis of AtPDI1 involved in the abiotic stress response. [file 12870_2021_3325_MOESM6_ESM.docx]
